# Characterization of Wall Paintings of the Harem Court in the Alhambra Monumental Ensemble: Advantages and Limitations of In Situ Analysis

**DOI:** 10.3390/molecules27051490

**Published:** 2022-02-23

**Authors:** Paz Arjonilla, Ana Domínguez-Vidal, Ramón Rubio Domene, Elena Correa Gómez, María José de la Torre-López, María José Ayora-Cañada

**Affiliations:** 1 Department of Physical and Analytical Chemistry, Faculty of Experimental Sciences, Campus Las Lagunillas, Universidad de Jaén, E-23071 Jaén, Spain; mpurena@ujaen.es (P.A.); mjayora@ujaen.es (M.J.A.-C.); 2 Conservation Department, Council of the Alhambra and Generalife, E-18009 Granada, Spain; ramonf.rubio@juntadeandalucia.es (R.R.D.); elena.correa@juntadeandalucia.es (E.C.G.); 3 Department of Geology, EPSL, Campus Científico Tecnológico, Universidad de Jaén, Cinturón Sur s/n, E-23700 Jaén, Spain; mjtorre@ujaen.es

**Keywords:** wall painting, Alhambra, Raman spectroscopy, XRF spectroscopy, portable instrumentation, principal component analysis, stratigraphy

## Abstract

Non-invasive techniques (X-ray fluorescence, XRF, and Raman spectroscopy) were used for the study of the Hispano Muslim wall paintings. Principal component analysis (PCA) was performed on the semi-quantitative XRF results directly provided by the in-built factory calibrations with minimum user manipulation. The results obtained were satisfactory and highlighted differences and similarities among the measurement points. In this way, it was possible to differentiate the decorations carried out on gypsum plasterwork and the wall paintings over lime plaster. The color palette, revealed by combining the results from XRF and Raman spectroscopies, comprised the pigments hematite, lapis lazuli, cinnabar (in poor conservation state), and possibly, carbon. Evidence of past interventions was also provided by PCA on XRF data, which detected the presence of Pb, Ba, and Zn in some areas. Furthermore, the preparation layers have been studied in detail on cross-sections of two microsamples. Several layers of lime plaster with a compact microstructure have been observed. The characteristic of the pictorial layer and the identification of calcium oxalate point to the use of a secco-technique. The main alteration identified was a gypsum surface layer covering the painting and signs of plaster deterioration due to gypsum migration to more internal areas. Finally, the comparison with the observations made by restorers in previous interventions on these paintings revealed the importance of the representativeness gained with the in situ study, which enabled the analysis of a high number of areas.

## 1. Introduction

The Alhambra Monumental Ensemble at Granada (Spain) holds some of the few palaces to have survived from medieval Islamic times. This architectural complex, with its intricate succession of rooms and courts, gardens, fountains, and watercourses, is one of the most magnificent examples of Islamic architecture. It illustrates superbly the contrast between an unassuming exterior and a richly decorated interior. Nasrid arts grew from Almohad traditions, but displayed far more variety and splendor than their precursors. Tiling, together with carved and painted plasterwork, particularly in the form of *mocarabes* domes, are the most popular decorative revetments from Nasrid art. However, other less well-known architectural decorative solutions were also employed, as is the case with wall paintings. Mural painting in the Alhambra has a certain quantitative and qualitative entity, although it is not well known by most visitors. Most of these paintings are not part of the established circuits, probably due to their location in small rooms (like the paintings of the Partal house) and their precarious state of conservation [1]. The custom of painting walls with ornamental purposes can be traced back to earlier cultures. In Hellenistic times, decorative mural plastering was already used, a technique that survived through Roman art [2,3,4] with representative examples in the Iberian Peninsula [5]. Also in the Middle East, there was an important tradition of mural painting. Both precedents could have an influence on Hispano-Muslim mural painting. Wall painting in the Alhambra can be found in exterior walls and some other architectural elements like vaults, mainly in the form of faked breaking of bricks in red [6]. However, it stands out especially in the decoration of the wall socles, in rooms reserved for domestic activities and private places. Thus, in Nasrid architecture the tiled socles preferably occupy the official spaces of the palace, reserving the painted ones for more intimate rooms [1].

Most published studies report investigations regarding wall paintings have been carried out on fragments found in archaeological excavations [7,8,9,10], whereas studies on paintings preserved in their original locations are much scarcer [11,12,13]. However, these wall paintings are undoubtedly the most at risk due to their interaction with the surrounding environment. Therefore, the study of these artworks provides information not only about the original materials, but also about their interaction with the environment in which they are located [14]. Understanding the reasons for decay would aid in the design of subsequent adequate conservation procedures [15,16,17]. The use of mobile or portable systems for the characterization of materials in a non-invasive manner is particularly advantageous when dealing with wall paintings in their original locations [18,19,20]. New portable instruments developed and commercialized in the last two decades, enabled the direct measurements on artworks avoiding sampling [21,22,23,24,25]. In addition to the main benefit of not damaging the artwork, working in situ allows a more complete understanding of the architectural work under study than working with fragmentary samples in the laboratory. Spectroscopic techniques, especially, X-ray fluorescence (XRF), and Raman spectroscopy [20] are the most commonly used analytical techniques for in situ studies. XRF spectroscopy is a very valuable technique since it provides the elemental composition of the materials and generally is the technique of choice to first screen the artworks. The identification of inorganic key elements by in situ XRF can be already sufficient to guide conservation and restoration interventions, as shown in the study of wall paintings from the 14th and 15th centuries [26]. However, this simple approach, despite being the most commonly used, does not fully exploit the potential of the XRF technique. The current approach is moving to the incorporation of chemometrics tools to increase the amount of information that can be gathered with non-invasive XRF analysis. An attractive possibility is the use of non-pre-treated spectroscopic data, that is, the use of raw intensity data of emission lines with no calibration to concentrations [27]. A recent study has demonstrated the usefulness of this approach for grouping ancient pigments by color classes and elementary composition with validation on a real case of wall painting [27]. Nevertheless, handheld XRF commercial instruments fitted with factory in-built calibrations that provide direct conversion of the obtained spectra into elemental constituent concentrations without user interaction are proliferating. Most of these semi-quantitative determinations are based on mathematical algorithms such as Fundamental Parameters (FP) to correct for matrix effects [28,29]. These semi-quantitative results have been reported to lack enough analytical accuracy, particularly in the case of artworks, where the minimum requirements of sample homogeneity do not apply [29,30]. However, their usefulness to provide information of tendencies, groupings, and correlations will be here evaluated by using a multivariate exploratory tool as Principal Component Analysis (PCA). In any case, the elemental composition does not provide unambiguous identification of the material. Thus, Raman spectroscopy can be used to further confirm the identity of the compounds. The use of a portable or mobile Raman spectrometer connected to a micro-probe allows a non-destructive approach towards the work of art if the laser power is under control to avoid thermal degradation. Its reliability in the characterization of historical pigments, certain organic compounds, and degradation products is reported in many studies [20].

With the characterization of the wall paintings of the Harem Court in the Alhambra monumental ensemble, we contribute to the enlargement of the scarce information available about wall painting techniques in the Nasrid period. Furthermore, the work also critically assesses the information that can be gathered using portable analytical techniques for in situ non-invasive studies. In particular, the use of chemometric tools on XRF semi-quantitative data directly provided by factory calibrations is tested.

### Sites of Study

**Figure 1:**
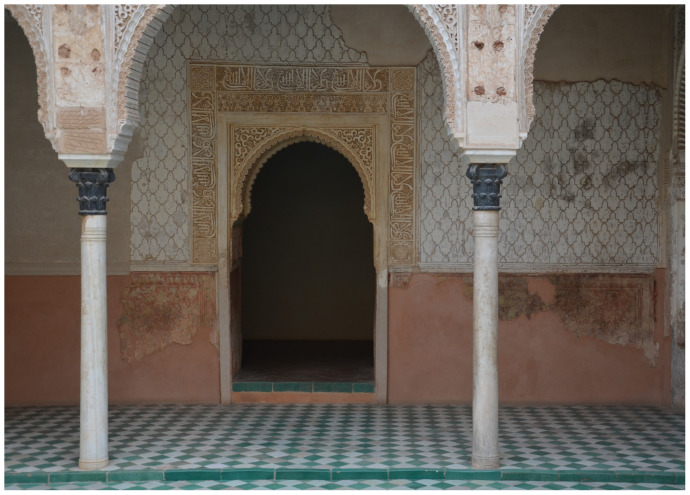
Photograph of the East portico in the Harem Court.

The Harem Court situated on the top floor of Abencerrages hall is one of the scarcely visited parts of the Palatine city of the Alhambra. It has two porticoes, which have three arches each (see Figure 1). The access is through a corridor illuminated by arches with latticework. The courtyard walls are decorated in white color with stripes, and the socles are painted in red, black, and blue with a geometric composition topped by a decorative border. A preparatory drawing over the finishing plaster is also observed. The painted socles of the portico date from the second half of the 14th century, corresponding to the reign of Muhammad V. The paintings possibly owe their preservation to the fact that they were covered with plaster immediately after the Christian occupation. In 1923, the restorer Torres Balbás [31] discovered the paintings and interpreted them as a whole in their relationship with the other elements of the court. His intervention filled the empty spaces with reddish plaster, keeping this filling at a lower level than the original paintings to emphasize them. Since this intervention, the paintings are more exposed to the environment and they have probably been restored repeatedly. The last documented intervention was carried out in 1988 by the conservation department of the Alhambra and Generalife Council [32].

## 2. Results

### 2.1. In Situ Study

A total of 40 measurement points in the court were characterized by XRF and Raman spectroscopy. They included locations of the different colors found in the mural paintings in the socles, in the decorative white plasterwork of the walls, and in the molding separating these two decorative elements. A detailed description of the measurement points can be found in the Section 3 and in Supplementary Material. Thirteen elements were detected by XRF, namely, Al, Ba, Ca, Cl, Fe, Hg, K, Pb, S, Si, Sr, Ti, and Zn. During the analysis of wall paintings by XRF, the fact that the volume of material analyzed will exceed the pictorial layer has to be considered. The depth of penetration of the primary X radiation is generally between 0.1 and 1 mm. However, the deepness from which the secondary radiation comes back to the detector is specific to each element and can vary depending on material density and matrix effects. In general, light elements are detected from more external layers than heavy ones.

**Figure 2:**
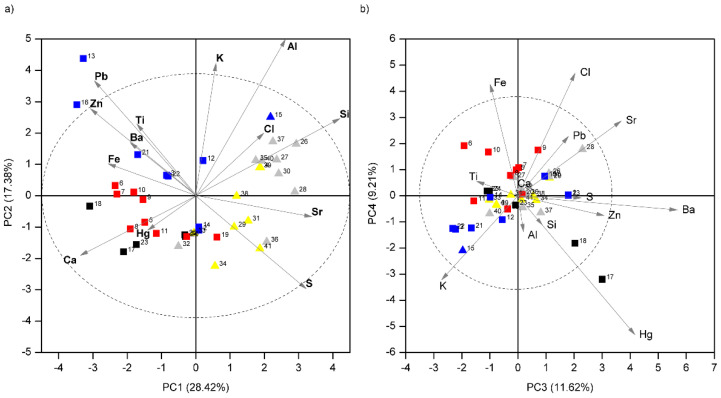
PCA results on XRF data. Biplots of scores and loadings for (**a**) PC1 versus PC2 and (**b**) PC3 versus PC4. Measurement points (scores) are labeled according to the color of the visual inspection and the support. Labelling: blue (■), red (■), and black (■) motifs in the socles; plasterwork substrate (▲) and white finishing layer (▲) in plasterwork.

Despite these difficulties for accurate quantification, data exploration of the semi-quantitative results directly provided by the factory calibration can be useful. These results are available in almost real-time and without any user manipulation. Thus, principal component analysis (PCA) was applied to the so-obtained concentration data to evaluate its potential to detect tendencies and groupings in the measurement points. Initially, models with raw data and autoscaled data were considered. Two principal components explained 99.74% of the cumulative variance (with PC1 accounting for 98.22%) for the model for the raw data (without autoscaling). The major contributions to both PCs were the two major elements detected, Ca and S. Both elements were identified in all measurements, independently of the color of the area, and therefore they presumably correspond to the underlying substrate. In comparison, autoscaling increased the importance of the elements present in the thin pictorial layer, reducing the weight of Ca and S. Therefore, only the results of the model with autoscaled data were further considered. Five principal components (with eigenvalues higher than 1) accounted for 75.03% of cumulative variance. The rather low percentage of variance captured by these PCs and the variance distribution among them revealed many different sources of variability in the dataset. The data pre-treatment also contributed to the complexity of the model, increasing the number of PCs to explain the total variance, since autoscaling often amplifies noise in the resulting data. This is due to inflation of the measurement error that is usually relatively large for small concentration values. Nevertheless, useful information can be extracted from the first four components of the PCA model (cumulative variance explained 66.63%), as shown in the biplots of Figure 2.

**Figure 3:**
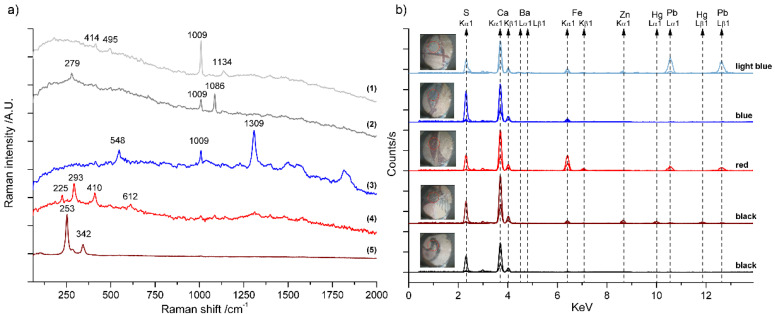
Representative spectra registered during the in situ study. (**a**) Raman spectra of different compounds identified in situ: (1) gypsum; (2) calcite with gypsum; (3) lapis lazuli; (4) hematite; (5) cinnabar. (**b**) XRF spectra (with filters for light, low and high ranges) of different motifs labeled with the macro-observed color. Inset images correspond to the analyzed area (marked with a red circle).

A clear distinction between XRF measurements on plasterwork and those on mural painting is observed along the axis defined by PC1 (28.42% of variance explained). Plasterwork samples (labeled with triangles in Figure 2) show positive scores values, whereas mural painting locations (labeled with squares) show mostly negative ones. Inspection of the loadings in the biplot revealed that the S and Sr contents are higher in the plasterwork motifs of the wall, while Ca is more abundant in the mural paintings of the socles. Thus, this component reflects the differences between the socle and the plasterwork substrates based on gypsum plaster and lime plaster, respectively. These findings are supported by Raman spectroscopy. As can be seen in Figure 3a, the spectral features of pure gypsum (CaSO_4_·2H_2_O, spectrum 1) were registered on plasterwork in the upper walls. The presence of Sr, and its abundance in the plasterwork, can be explained because it is a common impurity in natural gypsum. Sr can be either a substitute of Ca ions in the crystalline structure of gypsum or forming a different mineral phase, celestine (SrSO_4_). We also confirmed the presence of calcite (CaCO_3_) in different areas of the mural paintings regardless of the color by Raman spectroscopy (see Figure 3a). However, gypsum was also detected in these locations. The presence of calcite can be attributed to the underlying substrate based on lime plaster, whereas the presence of gypsum could be due to impurities of the lime, intentional addition or even degradation processes due to atmospheric contamination. This point is further clarified with the study of the stratigraphy of the decorations on sample cross-sections (see Section 2.2). Finally, it is interesting to note that Sr is a clue element in the PCA differentiation between locations on gypsum plasterwork (positive scores for PC1) and mural painting locations (negative scores for PC1). This is consistent with the fact that Ca substitution by Sr (with larger ionic radius) is not favored in the calcite structure [33].

Focusing on plasterwork locations, PC2 (17.38%) differentiates between measurements on the white finishing layer (negative scores) and those registered on the substrate below in areas where the white finishing was detached (positive scores). Looking at the loadings in the biplot, one can observe that the underlying substrate is richer in Si, Al, K, and Cl. This is consistent with the use of black gypsum, with aluminosilicate impurities for the plasterwork substrate and much purer gypsum (white gypsum) for the white finishing layer, as reported for other decorative plasterwork revetments in the Alhambra monument [34].

Considering the colors used in the mural painting, the most abundant is red. Looking at the PCA biplots (Figure 2), it can be observed that measurements on red motifs tend to group together. Raman spectra registered in the red motifs showed the characteristic signature of hematite (Fe_2_O_3_) with bands at 225, 292, 410, and 612 cm^−1^ (see Figure 3a) and are characterized by the presence of Fe in XRF (see Figure 3b). Other elements identified using XRF in variable amounts were Si, Al, and in minor proportion Ti. The identification of this hematite and the elemental composition are compatible with the use of red ochre pigments. These are commonly found in the wall paintings of the Alhambra complex, particularly in the characteristic Nasrid motifs imitating red bricks [6]. Red ochre has been also identified in other Islamic wall paintings in the Iberian Peninsula [35].

Regarding the blue motifs, the Raman spectra recorded in these areas presented a very characteristic pattern with a weak band at 548 cm^−1^, attributed to the symmetric stretching vibration of S^3−^, the chromophore responsible for the blue color of the mineral lazurite, and a series of more intense bands in the region 1250–1900 cm^−1^ (see Figure 3a). These bands are characteristic of natural ultramarine pigments and appear only when using 785 nm excitation [36,37]. They have been attributed to impurities present in the lapis lazuli rock from which the pigment was obtained [36,38]. The presence of this pattern of bands can be easily used to distinguish the natural pigment from the synthetic analogous, which started to be produced in the 19th century. This pattern has also been detected in other decorative revetments in the Alhambra, like marble capitals [39], wooden ceilings [40], and plasterworks [41,42]. When analyzing the PCA scores on the biplot (Figure 2a), the XRF measurements on blue motifs appear to spread, revealing a heterogeneous composition. For example, one of the measurements (M-15) was not performed on the socle, but on a blue motif painted on the plaster molding, which separates the socle from the upper wall. For this reason, this measurement appears together with those belonging to plasterwork in the scores plot, reflecting the influence of the underlying material on the elemental composition retrieved by XRF. More interesting is the case of measurement points 13 and 16, which appears out of the boundaries of the confidence ellipse of the PCA model. This means that their elemental composition is rather different from the rest. Looking at the loadings in the biplot, these differences can be mainly attributed to the presence of Pb and Zn, as can also be seen in the spectrum shown in Figure 3a (light blue). In fact, the blue motifs where these XRF spectra were registered showed a blue tonality lighter than the rest of the motifs. We initially attributed this fact to the partial loss of the pigment or its degradation because of previous aggressive interventions using acids [32] to remove the superficial contamination layers. However, the results of PCA point to the use of a mixture of the blue pigment with white pigments like lead white (2PbCO_3_·Pb(OH)_2_) and zinc white (ZnO) in these areas. Lead white was used as a pigment since antiquity and it has been found in the Alhambra complex in paintings on wood [40] and leather [43]. However, its association here with Zn, typical of modern white pigments, suggests a retouching [32]. Furthermore, a weak signal attributed to Ba was detected (see Figure 3b and Supplementary Material, Figure S2) and PCA results revealed a strong positive correlation between Zn and Ba in both biplots. This finding also points to the use of modern white pigments like lithopone, composed of a co-precipitate of zinc sulfide (ZnS) and barium sulfate (BaSO_4_). It was manufactured on a commercial scale at the end of the 19th century.

Finally, XRF spectra of five black motifs (a less abundant color in the paintings) were registered. Interestingly, in two of these locations, Hg was detected, as can be seen in the XRF spectrum of Figure 3a. In the biplot defined by PC3 and PC4 (Figure 2b), these points appear separated from the rest and the difference can be mainly attributed to the presence of Hg. Several Raman spectra were recorded in these black motifs and some of them showed the clear signature of cinnabar (see Figure 3b), an extremely strong Raman scatterer. This reveals that these black motifs were originally red and the pigment cinnabar has been degraded. The blackening of cinnabar has been observed on the surface of frescoes at important heritage sites such as Pompeii [44], and paintings from famous masters [45]. The degradation process is considered a complex phenomenon, still not completely understood. It has been reported to occur through different chemical pathways and with the participation of different alteration agents, like sunlight, alkalis, or chlorine ions [44,45,46]. Nowadays, it is widely accepted that chlorine plays a key role in the darkening process through the formation of light-sensitive mercury chloride compounds, or as a catalyst in the photochemical redox transformation of Hg(II)S into Hg(0) and S(0) [46]. In our previous studies in the Alhambra complex, we identified cinnabar mainly in the plasterwork decorations of the *mocarabes* vaults [42]. In these decorations, cinnabar was found in very different stages of conservation, ranging from its characteristic intense red tonality to grey and black shades. In that case, the identification of the Raman band of calomel (Hg_2_Cl_2_) proved the role of chlorine in the deterioration. Here, we did not identify calomel in the Raman spectra, but the quality of the spectra recorded in outdoor locations is not optimal for the detection of weak Raman signals. Nevertheless, XRF systematically detected the presence of low amounts of chlorine in the paintings of the court. Even if PCA does not show a correlation between Hg and Cl, its role as a catalyst in the degradation of cinnabar cannot be completely excluded.

The elemental composition retrieved by XRF in the other three black motifs analyzed did not reveal any characteristic element apart from those common to the substrate, which suggests the use of carbon-based pigments, as found in other Nasrid decorations in the Alhambra [41,42]. Here, Raman spectroscopy could not further confirm this point due to the poor quality of the spectra recorded in these motifs. Nevertheless, in these cases, black seems to be the original color employed by the artists.

### 2.2. Stratigraphic Study

The historical importance of these paintings and the scarce remains that are preserved discouraged us from taking a large number of samples that would cause excessive material and aesthetic damage. For this reason, only two samples were taken, one from a red decoration (R) and another one from the background on which the different geometric motifs were drawn (B).

**Figure 4:**
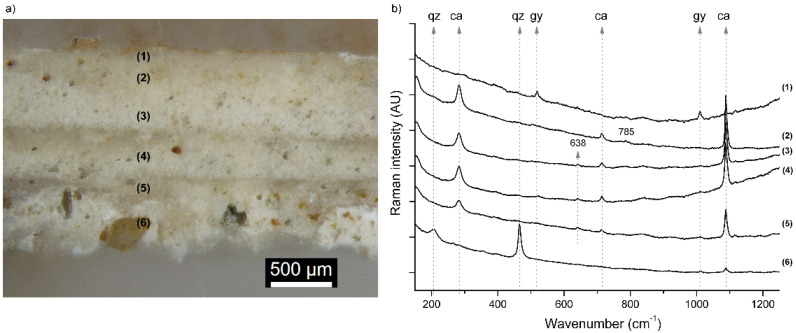
(**a**) Optical microscopy image of sample B and (**b**) Raman spectra recorded at different depths. Typical bands of qz: quartz; ca: calcite; gy: gypsum are marked. Excitation source: 785 nm; Objective 50×.

The microscopic observation of the sample taken from the background (B) reveals four different layers (see Figure 4a).

**Figure 5:**
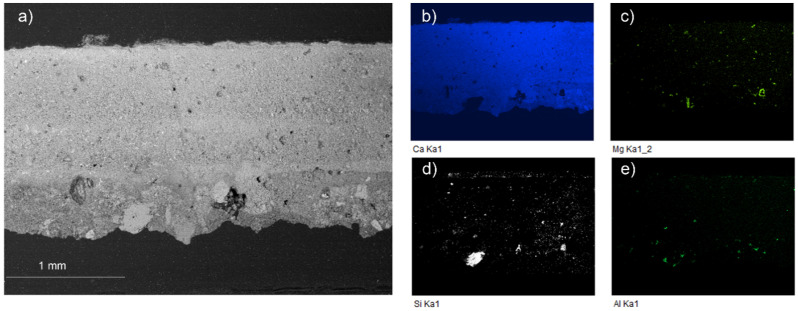
SEM-EDX results of sample B. (**a**) SEM image (BSE mode) and elemental maps of (**b**) calcium, (**c**) magnesium, (**d**) silicon, and (**e**) aluminum.

The deepest layer, applied to equalize the wall substrate, shows coarser grains of different minerals. Over this first layer, there are two more preparatory layers of very fine white plaster. The grain size in these layers is smaller than in the deepest layer, where some grains even reach 200 μm. Over these preparatory layers, there is a golden-yellowish discontinuous superficial layer that could correspond to the remains of former surface treatment. SEM images reveal a compact structure with very small pores in the preparatory layers. EDX analyses showed a high amount of calcium in these layers (see Figure 5), present in the form of calcite (CaCO_3_) as revealed by Raman spectroscopy (see Figure 4b). Few Mg impurities were also detected. The deepest layer was richer in inert aggregates of quartz and aluminosilicates, as can be seen in the elemental mapping of Si and Al in Figure 5. α-Quartz grains were identified by Raman spectroscopy by the sharp and strong mode at 464 cm^−1^ and another minor band at 205 cm^−1^ (Figure 4b), whereas the weak signals from aluminosilicates (like those observed at 638 and 785 cm^−1^) hindered their detailed identification. The high fluorescence of the Raman spectra registered from the outer golden-yellowish discontinuous layer and the EDS results suggest an organic nature. Likewise, Medina-Flórez [47] reported that glue was applied by restorer-architect Torres Balbás to fix the pigments, thus this could be the origin of this layer.

**Figure 6:**
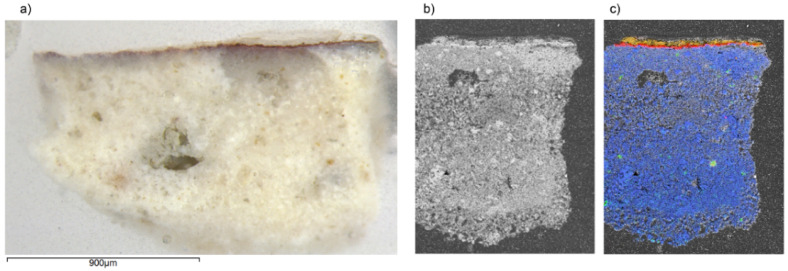
(**a**) Optical microscopy image of sample R. (**b**) SEM image of sample R (BSE mode) and (**c**) SEM-EDX results of sample R: superposition of elemental maps of calcium (blue), iron (red), sulphur (yellow), and silicon (green).

**Figure 7:**
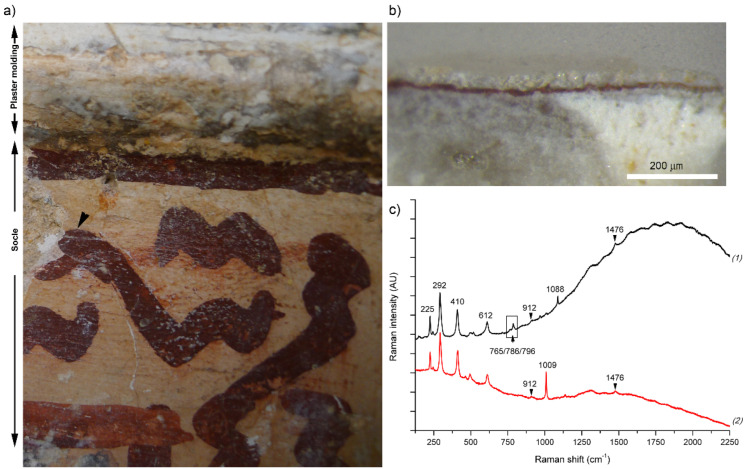
(**a**) Image showing the location where sample R was taken. (**b**) Optical microscopy image of sample R, focusing on the pictorial layer. (**c**) Raman spectra recorded (excitation 785 nm; objective 50×) on the pictorial layer of sample R: (1) internal part of the pictorial layer and (2) external part.

The microscopic observation of the stratigraphic cross-section of the sample of red painting (R) revealed a reasonably well preserved pictorial layer with a medium thickness of about 5 µm. Over the pictorial layer, there is another white translucent external layer. The pictorial and preparation layers seem to be well separated, which suggests a *secco* technique [48]. Elemental point spectra and two-dimensional (2D) elemental mapping by SEM-EDS showed the presence of Fe in the red pictorial layer associated with Al and Si in a lower amount (see Figure 6). Raman spectroscopy also confirmed the presence of hematite (see Figure 7). The narrow bandwidths observed and the absence of a band at 660 cm^−1^ (attributed to a prohibited Raman mode, which appears due to the breaking of symmetry induced by disorder) indicate good crystallinity [49,50]. In addition, the group of weak Raman bands at 796, 786, and 765 cm^−1^ can be attributed to biotite. They correspond to the vibrational modes of Si-Ob-Si bonds (Ob = bridging oxygen), which connect the SiO_4_ tetrahedra that makeup phyllosilicate layers [51]. These findings are in agreement with the results of the in situ study and reveal the use of a red ochre pigment very rich in well-crystalized hematite. The high fluorescence backgrounds in most Raman spectra registered in the pictorial layer suggest the use of an organic binder. Furthermore, weak Raman signals detected at 912 and 1476 cm^−1^ (see Figure 7) can be attributed to calcium oxalate in the form of weddellite (CaC_2_O_4_·2H_2_O). The presence of this compound can be interpreted as the result of organic binder degradation [52]. Weddellite was detected, especially when focusing on the exterior areas of the pictorial layer, more exposed to the environment. Gypsum was also identified within the pictorial layer (see Figure 7), being particularly abundant when focusing on the external side. Regarding the preparation layer, it was mainly composed of calcium carbonate in the form of calcite, as in the sample taken from the background, but SEM images revealed a less compact structure in the preparation layers. Furthermore, the main difference in the chemical composition is the presence of S in this sample, identified as gypsum by Raman spectroscopy. EDX also revealed the presence of Sr impurities, allocated together with S, which points to the presence of celestine (SrSO_4_). However, no clear Raman features of this compound were detected, which suggested that Sr substitutes for Ca in gypsum structure. Gypsum was also the main component identified in the most external layer over the red pigment. In this case, it could be attributed to rests left after removing the layer of gypsum plaster applied over the paintings in the past. In the case of the preparation layers, the absence of gypsum in the lime plaster of sample B discards the possibility of an intentional addition to reducing the setting time. It could be due to impurities of the lime plaster. However, considering the location where this sample was taken (very close to the plaster molding, which separates the socle from the upper wall, see Figure 7a), another possibility is the migration of the more soluble gypsum from the molding and the upper wall. High moisture levels associated with exposition to rainfall and poor maintenance in the past may have facilitated the migration process by water diffusion. This has contributed to a certain deterioration of the calcite binder as reflected in the SEM image.

### 2.3. Comparison with Observations Made by Restorers in Previous Interventions and Critical Assessment of Information Gained in the In Situ Study

These results are only partially in agreement with the observations made in 1988 by the restorers of these paintings and their study of the stratigraphy of a limited number of samples [32,47]. Concerning the pigments, they identified iron oxide and lapis lazuli, coinciding with our results, but they did not find cinnabar, probably because of its poor conservation state and the limited number of samples. In addition, the presence of the blue pigment azurite was stated, although it is not completely clear if it was found in the paintings of the Harem Court or at another location included in their study (a room annex to Sala de la Barca). Our results, with a complete and representative non-invasive analysis of the blue motifs, definitely discard the presence of this pigment in this location since no Cu was detected. The light blue tonality observed in certain motifs, that could resemble the color of azurite, must be attributed to the use of lead white in previous non-documented interventions. Furthermore, according to these reports, the support of the paintings was a coarse gypsum-rich lime mortar, covered with a thinner layer of lime plaster much finer and purer. The pigments were applied over this finishing layer using an organic binder [32,47]. Our results show that the support of the paintings is a carefully manufactured lime plaster with few impurities. No evidence of the use of organic additives has been found in this layer with FT-IR microscopy (only the typical bands of calcite were observed, data not shown), which contradicts the observations previously reported. The coarser mortar in the deepest layers contains more aggregates (quartz and phyllosilicates) but not gypsum. As discussed above, the presence of gypsum is related to plaster showing more porosity and holes and must be due to contamination/migration from the gypsum plaster present on the upper wall and/or covering the paintings. Furthermore, the thin translucent layer over the paintings described in the restoration report as “carbonation” is not calcium carbonate but gypsum, probably rests of the plaster covering the paintings during centuries, although a certain contribution of atmospheric contamination cannot be completely excluded. Our results also contradict some of the statements reported by Rallo Gruss [1] in a complete work on the origin and evolution of medieval Muslim wall painting. In that work, the last layer (preparation layer) is considered a gypsum plaster, and azurite is supposed to be present as the blue pigment, statements discrepant with the here-presented results.

This study also demonstrates the great potential of the non-invasive approach. With the in situ study, it was possible to analyze a large number of points achieving more representativeness. The use of two portable instruments providing elemental and molecular information has allowed the unambiguous identification of most of the pigments employed. However, the signal obtained, depending on the technique used, can provide information that is related not only to the surface of the object, but also to the underlying layers, complicating the interpretation. As it has been shown, the use of chemometric approaches to highlight correlations and groupings can further aid in the interpretation. In this way, the elemental information provided by XRF can be used more efficiently than with the common but limited approach of just identifying key elements to infer the presence of certain pigments. Nevertheless, results are more complete when portable techniques are supported by laboratory analyses on selected samples to achieve detailed stratigraphic information. Thus, the optimal solution is a multimodal approach based on the combination of a first noninvasive step followed by a focused sampling relying on the data acquired during the first step.

## 3. Materials and Methods

### 3.1. Methodology for Non-Invasive In Situ Analysis

The selection of the measurement locations was based on the visual examination of the colors and tones of the paintings. Up to 40 different points were considered for analysis, including the socles (where the majority of polychrome geometric motifs were found), the upper walls, and the plaster molding in between. They comprised all the colors observed (red, 9; blue, 9; black, 5). Furthermore, eight points in the white finishing of the upper wall and nine of the underlying substrate exposed in damaged parts of the wall were also considered. A detailed description of the measurement locations can be found in Supplementary Material.

Elemental analysis was performed on-site using a hand-held Niton^®^XL3t GOLDD+ X-ray fluorescence (XRF) spectrometer with a silver anode (50 kV, 200μA) (Thermo Fisher Scientific, Waltham, MA, USA). The analyzer is fitted with a camera, allowing closer visualization of the measurement area that can be set to either 8 or 3 mm diameter by collimation of the primary beam. Considering the characteristics of the motifs analyzed the smallest spot available (3 mm) was used. Spectra were collected using the measuring mode “mining”. The Niton XL3t GOLDD+ Analyzer is equipped with different excitation filters that optimize the analyzer’s sensitivity for various elements. The *Main range* filter provides good sensitivity from Mn to Bi. The *High range* and the *Low range* filters optimize detection from Ba to Ag, and from Ti to Cr, respectively. The *Light range* filter is used for light element analysis (down to Mg). Measurements of 20 s for each filter were set, which means that each spot analysis took about 80 s to be completed. NITON Data Transfer (NDT©) software was used to control the instrument and for data management and transfer. The internal pre-set calibrations based on Fundamental Parameters provided semi-quantitative concentration data for the different elements detected. The Fundamental Parameters approach relies on the mathematical description of instrumental conditions (e.g., tube emissions, detector efficiency) and instrument-independent parameters (e.g., fluorescence intensities, absorption coefficients, absorption edges). It uses X-ray theory to mathematically pre-determine inter-element matrix effects, thus it is useful to analyze samples of unknown chemical composition, in which the concentrations of light and heavy elements can vary from mg/kg to a high percentage. The concentration values from the detected elements and those for the non-detected light elements (estimated considering the Compton peak in the fluorescence spectra and labeled as ‘balance’ by the Niton analyzer) are summed to 100%. Raman spectra were recorded on-site using a mobile innoRam spectrometer (B&W TEK Inc., Newark, NJ, USA). It was equipped with a 785 nm laser for excitation, a CCD detector thermoelectrically cooled to −20 °C, and a fiber optic probe. The probe was coupled to a video-microscope sampling system with an integrated camera and a long focal distance objective (20×). The video microscope head was attached to an accessory motorized in the X-Y-Z axes with remote control for positioning and focusing. The laser power was limited to a few milliwats during spectra acquisition to avoid any damage to the materials. Other instrumental parameters like exposition time and the number of accumulations were optimized for each measurement depending on the sensitivity of the signal, fluorescence effects, etc. Typical acquisition conditions were 20 mW of nominal laser power, 10 s of exposition time, and 5 accumulations. The Raman shift covered is from 65 to 2565 cm^−1^ and the spectral resolution is about 2 cm^−1^.

### 3.2. Sample Preparation

Two samples were selected to further study the stratigraphy of the paintings (location can be found in Supplementary Material). First, a Leica M205C stereo-microscope (Leica Microsystems, Wetzlar, Germany) equipped with a Leica DFC450C digital camera was employed to observe the morphology of the samples. Then, the samples were embedded in a polyester resin and cut with a diamond saw blade to obtain cross-sections. The so-prepared cross-sections were observed and photographed again using the stereo-microscope and sequentially studied by Raman microspectroscopy, infrared spectroscopy, and SEM-EDX.

### 3.3. Laboratory Analytical Techniques

A Renishaw inVia Qontor Raman microspectrometer (Resnishaw plc., Wotton-under-Edge, UK) was used to register Raman spectra and images. Three excitation lasers were available namely, green (532 nm), red (633 nm), and one in the region of near-infrared (785 nm). Different objectives (5×, 20×, 50×, or 100×) were used to visualize and focus the laser on the sample. The acquisition conditions were adapted to a compromise between good signal-to-noise ratio and to prevent any sample damage.

Scanning electron microscopy was performed by using a Carl Zeiss MERLIN (FESEM) (Carl Zeiss AG, Oberkochen, Germany) equipped with BSE and SE detectors to obtain both secondary electron (SE) and retro-dispersive electrons (BSE) images. Elemental microanalysis was also performed for elements with Z > 4, with an energy dispersive X-ray spectroscopy detector EDX, Oxford Inca Energy 350X-MAX 50, and a WDX spectrometer, Oxford Inca Wave 500 (Oxford Instruments, Abingdon, UK).

### 3.4. Statistical Approach

The software SOLO + MIA (release 9.0) from Eigenvector Research Inc. (Manson, WA, USA) was used to perform Principal Component Analysis. This exploratory tool was used to extract information from the elemental semi-quantitative data provided by the in-built factory calibrations of the XRF instrument. Concentration data were preprocessed by autoscaling, that is, mean-centered and divided by standard deviation.

## 4. Conclusions

A complete in situ study has been performed on the decorative elements of the Harem Court, focusing mainly on the painted socles and also considering the white finishing of the upper walls. The non-invasive approach using XRF and Raman spectroscopy enabled the identification of the pigments employed, namely, hematite, lapis lazuli, cinnabar, and possibly, carbon. Furthermore, the suitability of using the semi-quantitative concentration data obtained from the XRF analyzer without user manipulation has been demonstrated. The results of PCA on the semi-quantitative data revealed differences between different substrates (lime plaster/gypsum plasterwork) and highlighted the presence of elements characteristic of modern pigments (Zn, Ba) in certain areas. The complementary study on cross-sections provided further information about the stratigraphic succession of layers, paint layers thickness and composition, pictorial technique, and, finally, its state of conservation. Our results contradict many aspects of earlier findings on these paintings, retrieved from studies on a very limited number of samples and with limited analytical techniques, highlighting the importance of the use of non-invasive in situ studies to guarantee representativeness.

## Data Availability

Data available.

## Supplementary Materials

The following supporting information can be downloaded online, Figure S1: Location of the measurement points (2–41) and samples (R and B) in the Harem Court, Figure S2: Zoom in the XRF spectra (obtained using the low filter) to show the smallest contributions: (a) in a black motif (measurement point 24) and (b) in a blue motif (measurement point 16), Table S1: Description of the measurement points.
