# Supplementary material for: Characterization of Wall Paintings of the Harem Court in the Alhambra Monumental Ensemble: Advantages and Limitations of In Situ Analysis
Source: Molecules. 2022 Feb 23;27(5):1490. doi: 10.3390/molecules27051490 (PMC8912083; doi:10.3390/molecules27051490)

Manuscript ID: **molecules-1574157**
Article type: Article
Title: Characterization of wall paintings of the Harem Court in the Alhambra monumental ensemble. Advantages and limitations of in situ analysis
Journal: *Molecules*
Section: Cross-Field Chemistry
Special Issue: Pigments in Wall Painting: An Archaeometric Study

Supplementary Material

Figure S1. Location of the measurement points (2-41) and samples (R and B) in the Harem Court.

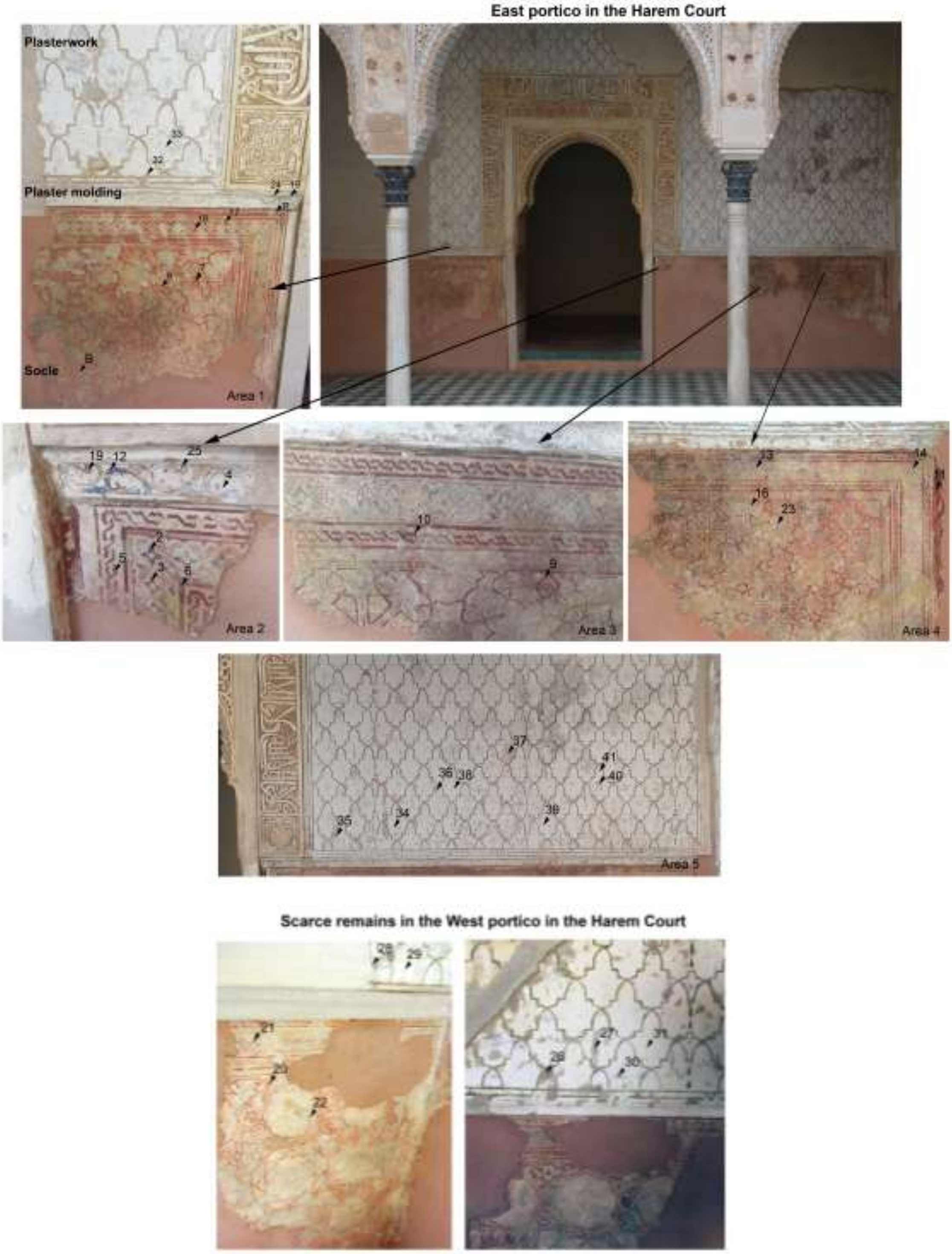

Manuscript ID: **molecules-1574157**
Article type: Article
Title: Characterization of wall paintings of the Harem Court in the Alhambra monumental ensemble. Advantages and limitations of in situ analysis
Journal: *Molecules*
Section: Cross-Field Chemistry
Special Issue: Pigments in Wall Painting: An Archaeometric Study

Table S1. Description of the measurement points.

Point	Observed color/material	Location	Typology
**1**	**System calibration**	-	-
**2**	blue	East portico - Area 2	Socle
**3**	blue	East portico - Area 2	Socle
**4**	white	East portico - Area 2	Plaster molding
**5**	red	East portico - Area 2	Socle
**6**	red	East portico - Area 2	Socle
**7**	red	East portico - Area 1	Socle
**8**	red	East portico - Area 1	Socle
**9**	red	East portico - Area 3	Socle
**10**	red	East portico - Area 3	Socle
**11**	red	East portico - Area 4	Socle
**12**	blue	East portico - Area 2	Plaster molding
**13**	blue	East portico - Area 4	Socle
**14**	blue	East portico - Area 4	Plaster molding
**15**	blue	East portico - Area 1	Plaster molding
**16**	blue	East portico - Area 4	Socle
**17**	black	East portico - Area 1	Socle
**18**	black	East portico - Area 1	Socle
**19**	red	East portico - Area 2	Plaster molding
**20**	red	West portico - Area 6	Socle
**21**	blue	West portico - Area 6	Socle
**22**	blue	West portico - Area 6	Socle
**23**	black	East portico - Area 4	Socle
**24**	black	East portico - Area 1	Plaster molding
**25**	black	East portico - Area 2	Plaster molding
**26**	gypsum	West portico - Area 7	Plasterwork
**27**	gypsum	West portico - Area 7	Plasterwork
**28**	gypsum	West portico - Area 6	Plasterwork
**29**	white	West portico - Area 6	Plasterwork
**30**	gypsum	West portico - Area 7	Plasterwork
**31**	white	West portico - Area 7	Plasterwork
**32**	gypsum	East portico - Area 1	Plaster molding
**33**	white	East portico - Area 1	Plaster molding
**35**	gypsum	East portico - Area 5	Plasterwork
**36**	gypsum	East portico - Area 5	Plasterwork
**37**	gypsum	East portico - Area 5	Plasterwork
**40**	gypsum	East portico - Area 5	Plasterwork
**34**	white	East portico - Area 5	Plasterwork
**38**	white	East portico - Area 5	Plasterwork
**39**	white	East portico - Area 5	Plasterwork
**41**	white	East portico - Area 5	Plasterwork

Note: A XRF spectrum was registered for each point. Several Raman spectra were also registered for each point, although not all of them contained useful information.

Manuscript ID: **molecules-1574157**
Article type: Article
Title: Characterization of wall paintings of the Harem Court in the Alhambra monumental ensemble. Advantages and limitations of in situ analysis
Journal: *Molecules*
Section: Cross-Field Chemistry
Special Issue: Pigments in Wall Painting: An Archaeometric Study

Figure S2. Zoom in the XRF spectra (obtained using the low filter) to show the smallest contributions: a) in a black motif (measurement point 24) and b) in a blue motif (measurement point 16).

a)

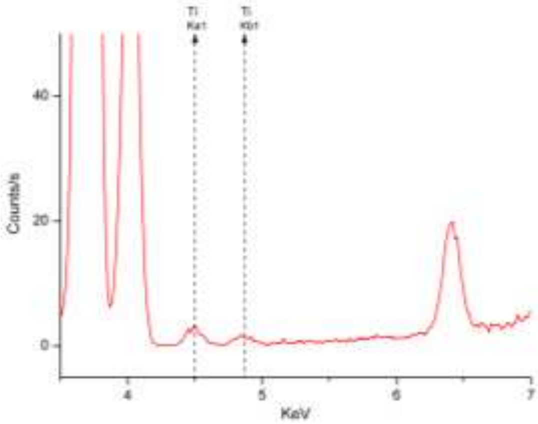

b)

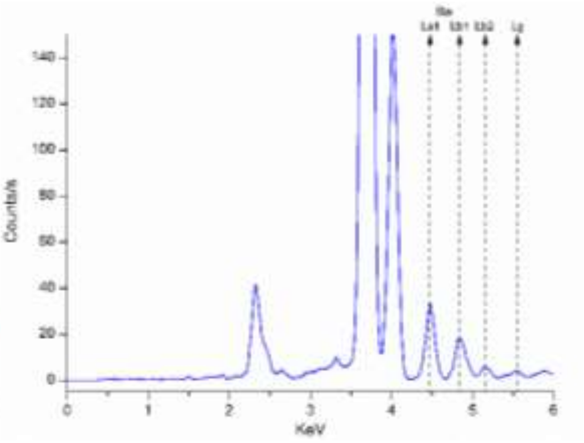

Supplement: Supplementary file 1 [file molecules-27-01490-s001.zip › molecules-1574157-supplementary.pdf]
